# Incidence and Short- to Intermediate-Term Oncological Outcomes of Pathological T0 Prostate Cancer After Robot-Assisted Radical Prostatectomy: A Multicenter, Retrospective Cohort Study in Japan (MSUG94 Group)

**DOI:** 10.3390/curroncol33060303

**Published:** 2026-05-22

**Authors:** Risa Tomioka-Inagawa, Masayuki Tomioka, Tomoyuki Tatenuma, Takeshi Sasaki, Yoshinori Ikehata, Akinori Nakayama, Masahiro Toide, Tatsuaki Yoneda, Kazushige Sakaguchi, Kazuhide Makiyama, Takahiro Inoue, Hiroshi Kitamura, Kazutaka Saito, Fumitaka Koga, Shinji Urakami, Takuya Koie

**Affiliations:** 1 Department of Urology, Gifu University Graduate School of Medicine, Gifu 501-1194, Japan; tomioka.risa.e8@f.gifu-u.ac.jp (R.T.-I.); tomioka.masayuki.e4@f.gifu-u.ac.jp (M.T.); 2 Department of Urology, Yokohama City University, Yokohama 236-0004, Japan; tatenuma@yokohama-cu.ac.jp (T.T.); makiya@yokohama-cu.ac.jp (K.M.); 3 Department of Nephro-Urologic Surgery and Andrology, Mie University Graduate School of Medicine, Tsu 514-8507, Japan; t-sasaki@med.mie-u.ac.jp (T.S.); tinoue28@clin.medic.mie-u.ac.jp (T.I.); 4 Department of Urology, University of Toyama, Toyama 930-0194, Japan; ikehatay@med.u-toyama.ac.jp (Y.I.); hkitamur@med.u-toyama.ac.jp (H.K.); 5 Department of Urology, Dokkyo Medical University Saitama Medical Center, Koshigaya 343-8555, Japan; akinori@dokkyomed.ac.jp (A.N.); kzsaito@dokkyomed.ac.jp (K.S.); 6 Department of Urology, Tokyo Metropolitan Cancer and Infectious Diseases Center Komagome Hospital, Tokyo 113-8677, Japan; masahiro_toide@tmhp.jp (M.T.); f-koga@cick.jp (F.K.); 7 Department of Urology, Seirei Hamamatsu General Hospital, Hamamatsu 430-8558, Japan; yonet@sis.seirei.or.jp (T.Y.); shinji.urakami@toranomon.gr.jp (S.U.); 8 Department of Urology, Toranomon Hospital, Tokyo 105-8470, Japan; sakaguchi-k@toranomon.gr.jp

**Keywords:** prostate cancer, radical prostatectomy, neoadjuvant hormonal therapy, pathological T0, biochemical recurrence

## Abstract

Background: Pathological T0 (pT0) prostate cancer following radical prostatectomy is uncommon, and its prognostic significance remains unclear, particularly after neoadjuvant hormonal therapy (NHT). We investigated the incidence of pT0 disease in a multicenter Japanese cohort and described postoperative biochemical recurrence (BCR) outcomes. Methods: This retrospective study analyzed 3079 patients who underwent robot-assisted radical prostatectomy at nine Japanese centers between 2011 and 2021. Patients were classified as having pT0 or non-pT0 disease. Because only four pT0 cases occurred without NHT, these are summarized descriptively. Exploratory Kaplan–Meier and log-rank analyses of biochemical recurrence-free survival (BRFS) were performed for the NHT subgroup. Results: Twenty-seven pT0 cases (0.9%) were identified, and 85.2% were identified after NHT. Overall, 399 patients (13.0%) developed BCR. Among patients who did not undergo NHT, the 1- and 2-year BRFS rates were 100% and 100%, respectively, in the pT0 group and 92.4% and 88.1%, respectively, in the non-pT0 group. In the NHT subgroup, the corresponding rates were 92.9% and 92.7%, versus 91.8% and 85.5%, respectively (*p* = 0.651). Conclusions: pT0 disease after robot-assisted radical prostatectomy is rare and occurs predominantly after NHT. Given the possibility that late-onset recurrences may have been overlooked, the results of this trial should be understood as providing evidence from the short- to intermediate-term perspective.

## Simple Summary

Pathological T0 prostate cancer indicated that no residual tumor was found in the prostatectomy specimen despite a positive biopsy. This phenomenon remains poorly understood, particularly regarding whether the absence of cancer truly indicates a favorable outcome. We analyzed 3079 Japanese men who underwent robot-assisted prostate removal at nine medical centers. Only 27 patients (0.9%) had pathological T0 disease, 23 of whom had received neoadjuvant hormonal therapy (NHT). Four patients had pathological T0 disease without NHT, and none developed biochemical recurrence during the limited follow-up period. In the NHT subgroup, pathological T0 was associated with numerically favorable 2-year biochemical recurrence-free survival, although the analysis was exploratory because only two events occurred in the pathological T0 group. These findings indicate that pathological T0 after radical prostatectomy is rare in Japan and should be interpreted with caution, especially when it follows NHT. Given the possibility that late-onset recurrences may have been overlooked, the results of this trial should be understood as providing evidence from the short- to intermediate-term perspective.

## 1. Introduction

Prostate cancer (PCa) remains a major public health concern worldwide. According to the National Cancer Center of Japan, prostate cancer was the most frequently diagnosed cancer among Japanese men in 2021, and the lifetime risk of diagnosis in Japanese men is 10.9% [1]. Globally, prostate cancer accounted for approximately 1.47 million new cases and 397,000 deaths in 2022, making it the second most common cancer in men [2]. Pathological stage T0 (pT0) prostate cancer is defined as the absence of residual carcinoma in a radical prostatectomy specimen after a previously positive biopsy [3], a phenomenon often described as vanishing prostate cancer [4].

The incidence of pT0 is extremely rare, occurring in 0.1–1.3% of all radical prostatectomy (RP) cases without neoadjuvant hormonal therapy (NHT) [5,6,7,8]. Multiple plausible reasons are suggested for the absence of residual PCa in RP specimens, including complete removal of the tumor by diagnostic biopsy or transurethral resection of the prostate (TURP), misdiagnosis of benign tumors, and tumor disappearance with neoadjuvant therapy [3,9]. Several preoperative predictors of pT0 disease exist, including low preoperative prostate-specific antigen (PSA) levels, nonpalpable clinical T1 stage, low biopsy Gleason score, and a single positive biopsy core with a low tumor volume [3,6,10].

The prognostic value of pT0 was less certain when the NHT was introduced. Prior studies suggest that prolonged NHT can increase the likelihood of pT0, but postoperative biochemical recurrence still occurs in a subset of patients [3,11,12]. This question is clinically relevant in Japan because treatment response and disease characteristics may differ from those reported in Western cohorts [13,14]. Therefore, we evaluated the incidence of pT0 disease after robot-assisted radical prostatectomy in a Japanese multicenter cohort, described four patients with pT0 disease who did not receive NHT, and performed exploratory biochemical recurrence analyses in the NHT subgroup.

## 2. Materials and Methods

### 2.1. Patient Population

This multicenter retrospective cohort study included 3208 patients with PCa who underwent RARP at nine Japanese institutions between September 2011 and August 2021. This study was approved by the Institutional Review Board of Gifu University (approval number: 2021-B039, date: 4 August 2021) and the institutional IRBs of all participating centers. The requirement for informed consent was waived according to the Japanese ethical guidelines for research using existing data.

Preoperative age, height, weight, body mass index, serum PSA level, prostate volume, clinical stage according to the American Joint Committee on Cancer (8th edition) [15], biopsy Grade Group (bGG), National Comprehensive Cancer Network risk classification [16], Eastern Cooperative Oncology Group performance status, history of neoadjuvant therapy, serum albumin, hemoglobin, C-reactive protein levels, neutrophil, lymphocyte, and platelet counts, neutrophil-to-lymphocyte and platelet-to-lymphocyte ratios, and systemic immune-inflammatory index were recorded. The extent of lymph node dissection and nerve-sparing technique was determined at the surgeon’s discretion.

### 2.2. Pathological Analysis

Comprehensive histopathological evaluation of each prostatectomy specimen was performed at the participating institutions using whole-mount sections and the 2019 International Society of Urological Pathology framework [17]. According to general Japanese rules, the specimens were sectioned at ≤4 mm intervals. No central pathology review was conducted in this study, and data on additional leveling or immunohistochemical confirmation of candidate pT0 cases were not available.

### 2.3. Follow-Up Schedule

Serum PSA levels were evaluated at 3-month intervals after RARP, with biochemical recurrence (BCR) defined as two consecutive postoperative measurements >0.2 ng/mL. When postoperative PSA levels did not decrease to <0.2 ng/mL, the RARP date was considered the time of BCR [18]. As this was a retrospective 9-center study spanning a decade, differences in visit timing and assay sensitivity across centers cannot be completely excluded.

### 2.4. Statistical Analysis

The patients were divided into pT0 and non-pT0 groups. Continuous variables were compared using the Wilcoxon rank-sum test, and categorical variables were compared using Fisher’s exact test or the chi-square test, as appropriate. Kaplan–Meier curves and log-rank tests were used only for exploratory analyses within the NHT subgroup because only four patients had pT0 without NHT. Sensitivity analysis repeated the exploratory survival analysis after excluding patients with a follow-up shorter than 12 months, unless BCR occurred earlier. All *p* values were two-sided, and *p* < 0.05 was considered statistically significant. Analyses were performed using R version 4.4.1 (R Foundation for Statistical Computing, Vienna, Austria).

## 3. Results

### Patients and Characteristics

**Figure 1:**
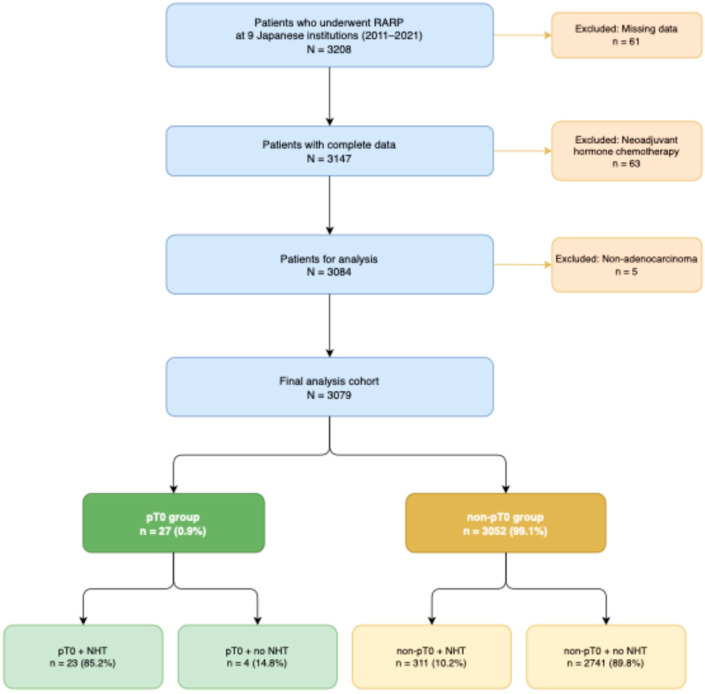
Patient flow diagram for cohort construction.

Of the 3208 enrolled patients, 61 with missing data and 63 who received neoadjuvant chemohormonal therapy were excluded. Subsequently, five patients with non-adenocarcinoma were excluded, leaving 3079 eligible patients for analysis (Figure 1).

Table 1 summarizes the clinicopathological characteristics of the patients. pT0 was identified in 27 patients (0.9%). Twenty-three of 334 patients (6.9%) who received NHT achieved pT0, whereas only four of 2745 patients (0.15%) who did not receive NHT achieved pT0. Pelvic lymph node dissection was performed in 2138 of 3079 patients (69.4%).

**Figure 2:**
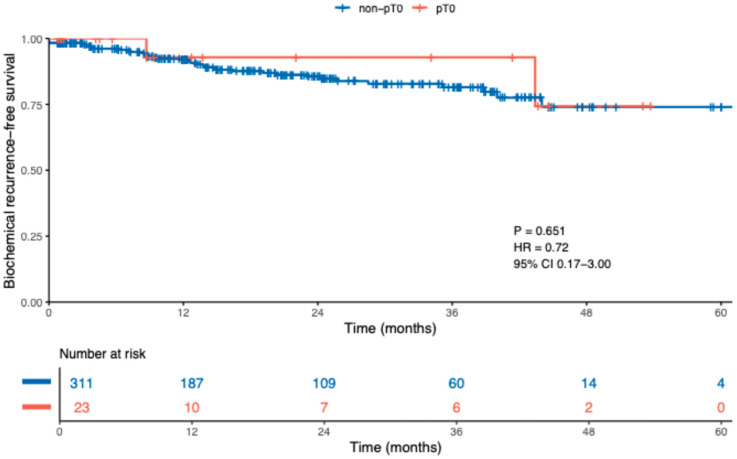
Kaplan–Meier estimates of biochemical recurrence-free survival for patients with NHT. One- and 2-year BRFS rates are 92.9% and 92.7% for pT0 and 91.8% and 85.5% for non-pT0, respectively (*p* = 0.651).

In the NHT subgroup (*n* = 334), the pT0 group included 23 patients and the non-pT0 group 311 patients, respectively (Table 2). Median follow-up was 9 months (IQR 2.5–37.5) in the pT0 group and 19 months (IQR 8–35) in the non-pT0 group. Two patients with pT0 disease and 39 patients with non-pT0 developed BCR. The 1-year and 2-year biochemical recurrence-free survival (BRFS) rates were 92.9% and 92.7% for pT0 and 91.8% and 85.5% for non-pT0, respectively (exploratory log-rank *p* = 0.651; Figure 2). In the sensitivity analysis restricted to patients with at least 12 months of follow-up or earlier BCR, 11 pT0 and 211 non-pT0 patients remained, and the 2-year BRFS estimates were 90.9% and 83.9%, respectively (*p* = 0.733).

The four pT0 cases without NHT are summarized in Table 3. Three patients had one or two positive biopsy cores, and one patient had incidental cancer detected during TURP. The follow-up period ranged from 1 to 81 months, and none of the patients met the study definition of BCR. By 25 months, only two patients remained under observation; therefore, these findings were descriptive only.

## 4. Discussion

This multicenter study showed that pT0 after robot-assisted radical prostatectomy was uncommon in Japan (27/3079, 0.9%) and occurred predominantly after NHT (23/27, 85.2%). Therefore, the overall pT0 rate was largely driven by the treatment effect rather than by the spontaneous disappearance of untreated low-volume cancer. In the NHT subgroup, pT0 showed numerically favorable short- to intermediate-term BRFS, but only two BCR events occurred in the pT0 group, and follow-up was shorter than in the non-pT0 group; therefore, these exploratory data should not be interpreted as showing equivalence, superiority, or durable long-term oncological safety.

The four patients with pT0 disease who did not receive NHT were best interpreted as a descriptive case series rather than as a comparison group. Their clinicopathological profiles were heterogeneous, and one patient had incidental cancer detected during TURP. No patient developed BCR, which is consistent with previous reports suggesting excellent outcomes in carefully selected untreated pT0 cases [10,19,20,21,22]. The reported prevalence of pT0 without NHT is similarly low, generally ranging from 0.1% to 1%, and has been linked to low-volume, low-grade disease [5,6,7,8,19,20]. Previous studies have suggested that pT0 is more likely in patients with limited cancer involvement on biopsy, such as a single positive core, very small tumor length, low grade disease, low PSA, and clinical T1c stage [3,7,10,19]. Possible explanations for untreated pT0 include the complete removal of a microcarcinoma at biopsy or TURP, limited residual tumor missed at pathological sampling, tissue loss during specimen processing, and diagnostic or specimen-handling issues. In our cohort, untreated pT0 cases were compatible with these mechanisms. However, the shortest observed follow-up in this subgroup was 1 month, and only two patients remained under observation beyond 24 months, precluding any inferential claim.

In the whole cohort, the pT0 group contained more high-risk and very high-risk National Comprehensive Cancer Network cases than the non-pT0 group because NHT was used mainly for high-risk disease. However, this pattern does not imply that biologically aggressive tumors are more likely to become pT0. Notably, the two recurrent pT0 cases after NHT had clinically advanced disease (cT3a), and one had bGG 5 and very high-risk classification, suggesting that baseline tumor aggressiveness can persist despite apparent pathological complete response [11,23]. Given that NHT may have been used exclusively in patients with highly aggressive PCa, its results should be considered limited.

Our findings should also be interpreted in the context of prior reports that prolonged NHT can increase the likelihood of pT0 without establishing a clear survival advantage [9,11,12,24,25]. The reported pathological complete response rates after NHT vary across studies, and a longer treatment duration, particularly beyond 6–8 months, has been associated with a higher chance of pT0 in some series [11,23]. However, randomized trials and meta-analyses have not demonstrated a clear long-term survival benefit, and current clinical guidelines do not recommend routine NHT for localized prostate cancer [16]. Japanese cohorts may differ from Western cohorts in host genetics, disease presentation, and treatment response [13,14,26,27]; however, the present data are insufficient to recommend a population-specific NHT strategy. Instead, the main clinical message is that pT0 should not be assumed to confer durable oncological safety after NHT. Although pT0 was achieved in 6.9% of patients treated with NHT, the types of drugs used, the duration of administration, and the clinical indications for NHT were not based on consistent criteria. Consequently, further investigation is necessary to determine the clinical significance of NHT and its true utility.

This study had several limitations. First, the follow-up period was substantially shorter in the pT0 group than in the non-pT0 group, and 14 of the 27 pT0 cases had less than 12 months of follow-up, creating a substantial risk of immature BRFS estimates. As prostate cancer recurrence may occur several years after surgery, late events may have been missed. Therefore, the 1-year and 2-year BRFS rates must not be considered definitive outcomes. Second, pT0 was rare, and only two BCR events occurred in the NHT pT0 group, which severely limited the statistical power. These results should perhaps be regarded as exploratory rather than as evidence of a long-term prognostic advantage derived from this trial. Third, NHT was recorded only as a binary exposure; the regimen, duration, and treatment interval were not available in this multicenter retrospective dataset. In addition, future research should examine whether the clinical indications for NHT and the implementation of NHT influence the incidence of pT0. Fourth, the pathological assessment was performed at each institution without a central review, and information on additional leveling or immunohistochemistry for candidate pT0 cases was unavailable. Furthermore, pathological processing protocols may differ between Japan and Western countries; under Japanese general rules, prostatectomy specimens are commonly sectioned at intervals of ≤4 mm, which may affect the detection of minute residual foci. Therefore, it is important to note that certain pT0 candidate findings may not accurately reflect complete pathological responses and could be a result of sampling errors. Fifth, the timing of postoperative visits and PSA assay sensitivity may vary across centers. Sixth, given the possibility that late-onset recurrences may have been overlooked, the results of this trial should be understood as providing evidence from the short- to intermediate-term perspective. Finally, the cohort was exclusively Japanese, which may limit its generalizability.

## 5. Conclusions

In this cohort, pT0 after robot-assisted radical prostatectomy was rare and observed mainly after NHT. The four patients with pT0 disease without NHT had no documented BCR; however, the subgroup was too small and immature for a formal comparison.

## Figures and Tables

**Table 1:** Clinicopathological characteristics in pathological T0 and other pathological T stages of prostate cancer patients.

Characteristic	pT0 Group N = 27	non-pT0 Group N = 3052	*p*
Age (years, median, IQR)	69 (66, 73)	68 (64, 72)	0.119
BMI (kg/m^2^, median, IQR)	24.4 (23.2, 27.5)	23.6 (21.8, 25.6)	0.059
Initial PSA (ng/mL, median, IQR)	10 (6, 15)	8 (6, 12)	0.246
Prostate volume (mL, median, IQR)	37 (25, 54)	30 (22, 40)	0.077
bGG (number, %)			0.004
1	5 (18.5)	611 (20.0)	
2	2 (7.4)	900 (29.5)	
3	4 (14.8)	616 (20.2)	
4	9 (33.3)	657 (21.5)	
5	7 (25.9)	268 (8.8)	
Clinical T stage (number, %)			0.401
1	4 (14.8)	571 (18.7)	
2	18 (66.7)	2165 (70.9)	
3	5 (18.5)	315 (10.3)	
4	0	1 (0.03)	
NCCN risk classification (number, %)			0.006
Low	1 (3.7)	316 (10.3)	
Favorable intermediate	4 (14.8)	585 (19.2)	
Unfavorable intermediate	3 (11.1)	1022 (33.5)	
High	16 (59.3)	999 (32.7)	
Very High	3 (11.1)	130 (4.3)	
Neoadjuvant hormonal therapy (number, %)	23 (85.2)	311 (10.2)	<0.001
PLND (number, %)	21 (77.8)	2117 (69.4)	0.345
Nerve-spare (number, %)			0.154
Not performed	24 (88.9)	2178 (71.4)	
Unilateral	3 (11.1)	671 (21.9)	
Bilateral	0	201 (6.6)	
Unknown	0	2 (0.07)	
Follow-up period (month, median, IQR)	11 (1, 41)	24 (11, 46)	0.010

pT0 Pathological T0, IQR Interquartile range; BMI Body mass index; PSA Prostate-specific antigen; bGG biopsy Grade Group; NCCN National Comprehensive Cancer Network; PLND Pelvic lymph node dissection.

**Table 2:** Clinicopathological features in pathological T0 and other pathological T stages of prostate cancer patients following NHT.

Characteristic	pT0 Group N = 23	non-pT0 Group N = 311	*p*
Age (years, median, IQR)	70 (66, 73)	69 (64, 73)	0.126
BMI (kg/m^2^, median, IQR)	24.7 (22.9, 27.6)	24.0 (22.2, 26.0)	0.260
Initial PSA (ng/mL, median, IQR)	9 (5, 14)	10 (7, 18)	0.211
Prostate volume (mL, median, IQR)	34 (25, 51)	29 (21, 40)	0.153
bGG (*n*, %)			0.747
1	3 (13.0)	21 (6.8)	
2	2 (8.7)	39 (12.5)	
3	3 (13.0)	46 (14.8)	
4	8 (34.8)	125 (40.2)	
5	7 (30.4)	80 (25.7)	
Clinical T stage (number, %)			0.494
1	2 (8.7)	29 (9.3)	
2	16 (69.6)	175 (56.3)	
3	5 (21.7)	106 (34.1)	
4	0	1 (0.3)	
NCCN risk classification (number, %)			0.576
Low	0 (0)	7 (2.3)	
Favorable intermediate	3 (13.0)	17 (5.5)	
Unfavorable intermediate	2 (8.7)	45 (14.5)	
High	15 (65.2)	193 (62.1)	
Very High	3 (13.0)	49 (15.8)	
PLND (number, %)	19 (82.6)	248 (79.7)	>0.999
Nerve-spare (number, %)			0.877
Not performed	20 (87.0)	265 (85.2)	
Unilateral	3 (13.0)	36 (11.6)	
Bilateral	0	10 (3.2)	
Follow-up period (month, median, IQR)	9 (1, 41)	19 (8, 35)	0.198

NHT Neoadjuvant hormonal therapy; pT0 Pathological T0; IQR Interquartile range; BMI Body mass index; PSA Prostate-specific antigen; bGG biopsy Grade Group; NCCN National Comprehensive Cancer Network; PLND Pelvic lymph node dissection.

**Table 3:** Clinicopathological characteristics of patients with pathological T0 without NHT.

Case	Age (Years)	Initial PSA (ng/mL)	bGG	Clinical T Stage	Biopsy Finding	Follow-Up (Months)	BCR
1	63	14.8	1	2a	2 positive cores	11	No
2	75	8.5	1	1c	2 positive cores	1	No
3	68	14.8	4	1b	Detected at TURP	81	No
4	65	11.7	3	2a	1 positive core	35	No

NHT Neoadjuvant hormonal therapy; PSA Prostate-specific antigen; bGG biopsy Grade Group; BCR Biochemical recurrence.

## Data Availability

The data presented in this study are available on request from the corresponding author. The data are not publicly available due to privacy and ethical reasons.

## Abbreviations

The following abbreviations are used in this manuscript:

BCR	Biochemical recurrence
bGG	Biopsy Grade Group
BMI	Body mass index
BRFS	Biochemical recurrence-free survival
IQR	Interquartile range
NCCN	National Comprehensive Cancer Network
NHT	Neoadjuvant hormonal therapy
PCa	Prostate cancer
PLND	Pelvic lymph node dissection
PSA	Prostate-specific antigen
pT0	Pathological T0
RARP	Robot-assisted radical prostatectomy
RP	Radical prostatectomy
TURP	Transurethral resection of the prostate
